# Structure and activation mechanism of the human liver-type glutaminase GLS2

**DOI:** 10.1016/j.biochi.2021.03.009

**Published:** 2021-06

**Authors:** Igor M. Ferreira, José Edwin N. Quesñay, Alliny CS. Bastos, Camila T. Rodrigues, Melanie Vollmar, Tobias Krojer, Claire Strain-Damerell, Nicola A. Burgess-Brown, Frank von Delft, Wyatt W. Yue, Sandra MG. Dias, Andre LB. Ambrosio

**Affiliations:** a Brazilian Biosciences National Laboratory, Brazilian Center for Research in Energy and Materials, Campinas, Sao Paulo, Zip Code, 13083-970, Brazil; b Sao Carlos Institute of Physics, University of Sao Paulo, Sao Carlos, SP, Zip Code, 13563-120, Brazil; c Graduate Program in Genetics and Molecular Biology, Institute of Biology, University of Campinas (UNICAMP), Campinas, São Paulo, Brazil; d Structural Genomics Consortium, University of Oxford, Old Road Campus, Roosevelt Drive, Headington, OX3 7DQ, UK; e Diamond Light Source Ltd., The Research Complex at Harwell, Harwell Science and Innovation Campus, Didcot, Oxfordshire, OX11 0FA, UK; f Department of Biochemistry, University of Johannesburg, Auckland Park, 2006, South Africa; g Research Complex at Harwell, Harwell Science and Innovation Campus, Didcot, OX11 0FA, UK

**Keywords:** Glutaminase, Liver-type, Isozyme, Breast cancer, Metabolism, Crystal structure, Kinetics, Cooperativity

## Abstract

Cancer cells exhibit an altered metabolic phenotype, consuming higher levels of the amino acid glutamine. This metabolic reprogramming depends on increased mitochondrial glutaminase activity to convert glutamine to glutamate, an essential precursor for bioenergetic and biosynthetic processes in cells. Mammals encode the kidney-type (*GLS*) and liver-type (*GLS2*) glutaminase isozymes. *GLS* is overexpressed in cancer and associated with enhanced malignancy. On the other hand, *GLS2* is either a tumor suppressor or an oncogene, depending on the tumor type. The GLS structure and activation mechanism are well known, while the structural determinants for GLS2 activation remain elusive. Here, we describe the structure of the human glutaminase domain of GLS2, followed by the functional characterization of the residues critical for its activity. Increasing concentrations of GLS2 lead to tetramer stabilization, a process enhanced by phosphate. In GLS2, the so-called “lid loop” is in a rigid open conformation, which may be related to its higher affinity for phosphate and lower affinity for glutamine; hence, it has lower glutaminase activity than GLS. The lower affinity of GLS2 for glutamine is also related to its less electropositive catalytic site than GLS, as indicated by a Thr225Lys substitution within the catalytic site decreasing the GLS2 glutamine concentration corresponding to half-maximal velocity (K_0.5_). Finally, we show that the Lys253Ala substitution (corresponding to the Lys320Ala in the GLS “activation” loop, formerly known as the “gating” loop) renders a highly active protein in stable tetrameric form. We conclude that the “activation” loop, a known target for GLS inhibition, may also be a drug target for GLS2.

## Highlights

• A positive cooperative mechanism of activation is demonstrated for the liver-type glutaminase.
• The novel crystal structure for the glutaminase domain of human GLS2 is presented.
• Site-directed mutagenesis suggest the activation loop as a hotspot for inhibitor design.

## 1 Introduction

Cancer cells undergo extensive reprogramming of the metabolic pathways related to energy and biosynthetic building block synthesis. In general, tumors differ from healthy cells in their utilization of glucose, typically secreted as lactate, even under conditions of normal oxygen tension, a phenomenon termed the “Warburg effect” [1]. However, in addition to glucose metabolism, increased glutamine utilization has also attracted the scientific community’s attention as a new target for the development of therapies [2]. In cancer, both glutamine uptake and the rate of glutamine-to-glutamate conversion are increased, with mitochondrial glutaminases playing central roles in this process [3,4]. Glutamine-derived glutamate is a key anaplerotic source of the tricarboxylic cycle, providing ATP and carbon species for biosynthetic pathways; also, glutamate participates in glutathione production and, therefore, cellular redox balance.

Mammals have two glutaminase genes. The *GLS* gene (position 2q32-q34 in the human genome), under the control of the MYC [4] and c-Jun [5] oncogenes, gives rise to the splicing variants glutaminase C (GAC) and kidney-type glutaminase (KGA) [6]. The *GLS2* (position 12q13.3 in the human genome) is a p53-inducible gene that uses alternative promotors to generate two isoforms: liver-type glutaminase (LGA) and glutaminase B (GAB) [7]. GLS2 was initially identified in the liver, but it is also found in brain tissues and the pancreas [8]. The first LGA cDNA was cloned from rat liver [9], and the first variant identified in human cells was cloned from ZR-75 lineage breast cancer cells and was then called the GA of the liver but is often also referred to as LGA [10,11]. However, the same research group that identified LGA in ZR-75 cells eventually renamed this glutaminase GAB and hypothesized that a shorter form of the enzyme (corresponding to rat LGA) might also exist in humans [12]. This hypothesis was subsequently demonstrated by the same group [7].

In contrast to the GLS isoforms GAC and KGA, GLS2 has been traditionally described as having high *K*_m_ (or high *K*_0.5_ for nonhyperbolic Michaelian behavior) for glutamine, low *K*_0.5_ for phosphate (although this ion induces attenuated activation), no inhibition by the reaction product glutamate, and an obligatory requirement for ammonia as an activator, which explains the sigmoidal shape of its kinetic curve [11,13,14]. As described above, aside from the longer GAB isoform, the GLS2 gene also codifies a shorter LGA isoform that is 67 amino acids shorter than GAB at the N-terminus. Additionally, in humans, LGA has two functional start codons, separated by 30 amino acids; only the longer version of the protein retains activity [15]. GAB is also active but seems to have different kinetics properties than those of LGA [10].

Glutamine metabolism and redox balance driven by GLS2 in the context of p53 activation is linked to its unique metabolic role in suppressing tumor growth [16,17]. Glioblastoma [18,19] and hepatocarcinoma [20], with restored *GLS2* expression, grow comparatively less aggressively. Equivalent data have also been presented for colorectal and non-small lung carcinomas [17]. On the other hand, MYCN-amplified neuroblastoma cells exhibit higher *GLS2* expression levels (compared to cells with nonamplified MYCN) and are particularly prone to apoptosis upon glutamine deprivation [21]. A series of small molecules with alkyl benzoquinone functional groups decreased the intracellular glutaminase activity in lung, breast, and liver carcinoma cell lines and inhibited GAB more strongly than KGA [22]. Finally, GLS2 was recently shown to be an oncogene in breast cancer in our laboratory [23] and by other groups [24]. These findings corroborate GLS2 as a potential anticancer target under specific contexts.

While several crystal structures of GLS are currently available and much is known about its mechanism of activity and inhibition [[25], [26], [27], [28]], little has been described about the structural determinants of GLS2, although we have determined the crystallographic structure of its C-terminal ANK domain [25]. The recent accumulated literature pointing to GLS2 as a previously unforeseen anticancer target makes the investigation of its mechanism of activity and opportunities for chemical inhibition a necessity. In this manuscript, we present the crystallographic structure of the human GLS2 catalytic domain and evaluate critical residues essential for its activity to provide a starting framework for the development of future GLS2-oriented therapies.

## 2 Results

### 2.1 At higher concentrations, GLS2 presents sigmoidal non-Michaelis-Menten kinetics

By using a construct in which the first 72 residues were deleted to form a sequence common in both LGA and GAB isozymes (herein called GLS2, UniProt reference sequence Q9UI32-1), we previously showed that GLS2 was a less active glutaminase isozyme (since it presented a higher *K*_m,_ lower *K*_cat_ and lower catalytic efficiency, *K*_cat_/*K*_m_, value), compared to GAC and KGA, when these were all assayed under identical protein and phosphate (Pi) concentrations [26].

**Fig. 1:**
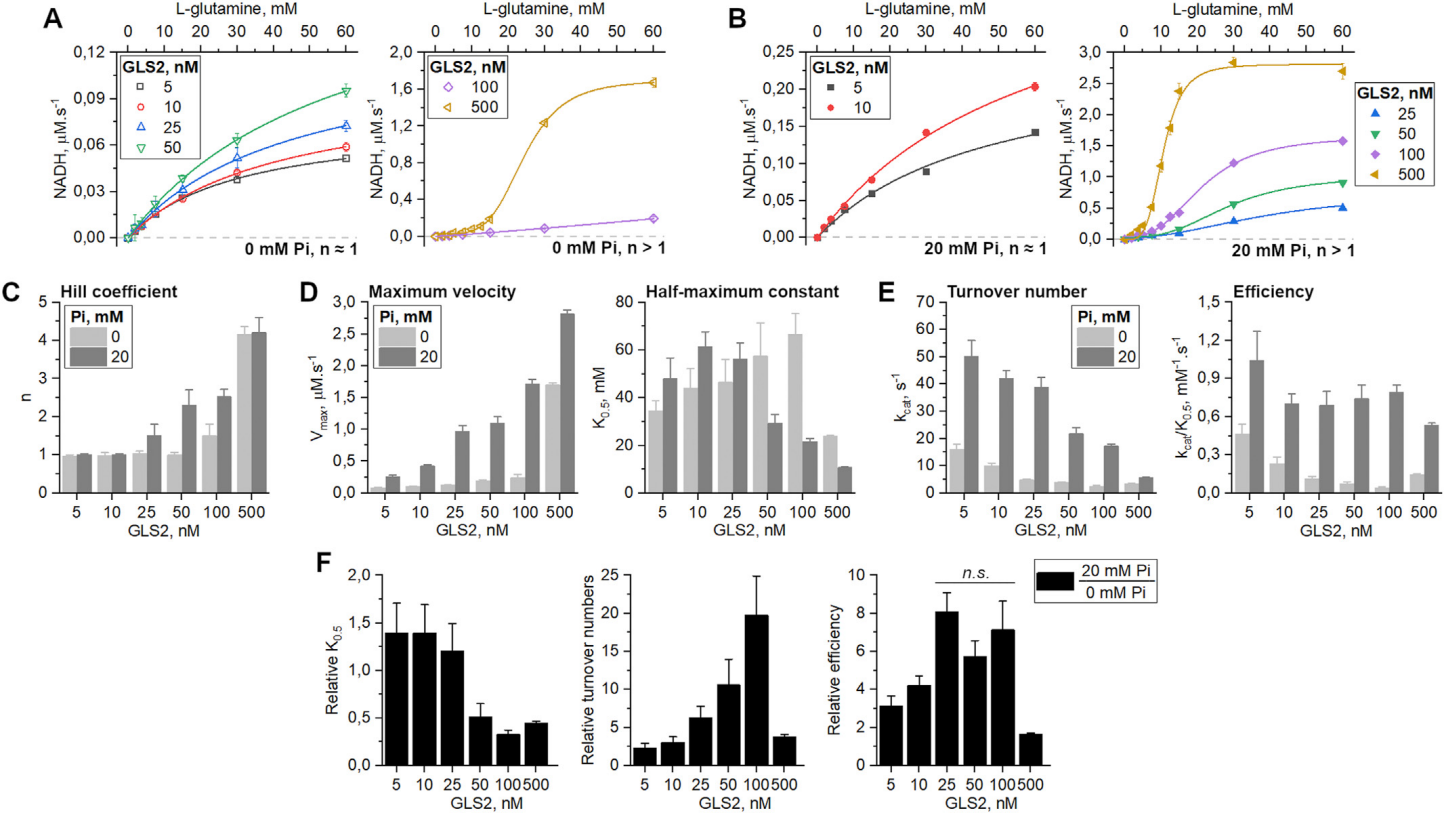
**Enzymatic characterization of GLS2.** The allosteric cooperative activation profile of GLS2 is dependent on the protein concentration **(A)** and is amplified in the presence of 20 mM inorganic phosphate **(B)**. **(C)** Increasing protein concentration and phosphate levels increase the Hill coefficient, calculated based on the sigmoidal kinetic curves up to ∼4. (**D**) Vmax and K_0.5_ of GLS2 at increasing protein concentrations in the absence or presence of phosphate and catalytic efficiency, followed by respective **(E)** turnover rates (k_cat_ = Vmax.[GLS2]) and efficiency (k_cat_.K_0.5_^−1^). (F) Relative kinetic parameters calculated as the ratio between the presence and absence of inorganic phosphate. *n.s.* means statistically non-significant, according to a multivariate ANOVA.

In the present work, we performed a detailed enzymatic study of mouse GLS2 to evaluate protein concentration and phosphate’s combined effect on its oligomerization and activity. The reaction velocities driven by GLS2 (from 5 nM to 500 nM protein concentration) in the absence or presence of 20 mM Pi were measured against and plotted as a function of increasing amounts of l-glutamine (up to 60 mM). The resulting curves can be clustered into two main categories according to the refined value of *n* after model fitting with a 4-parameter Hill equation. More specifically, the graphs on the left in Fig. 1A and B shows that, for the lower GLS2 concentrations, i.e., between 5 and 50 nM in the absence of Pi, and 5 and 10 nM with 20 mM Pi, *n* is approximately 1.0 ± 0.1 (Fig. 1C). This indicates that noncooperative Michaelis-Menten-like hyperbolic kinetic models best explain the data; in this situation, *K*_*0.5*_, or the substrate concentration for half-saturation, is equivalent to K_m_. However, at higher protein concentrations, *n* increases to be approximately equal to 4 (Fig. 1C) at 500 nM GLS2. In this situation, the curves are explained by a sigmoid, indicating positive cooperativity (Fig. 1A and B, graphs on the right). Interestingly, this shift in the enzymatic kinetic profile coincides with a change in the protein’s oligomeric state, transitioning from dimer to tetramer, as the protein concentration increases in the absence of phosphate (Fig. 3D).

Phosphate stimulates a complete transition in quaternary structure toward tetramerization (Fig. 3d), even at the lowest GLS2 concentration, namely 5 nM. As the protein concentration rises further, a continuous increase in maximum reaction velocity (V_max_), both with and without phosphate, is observed (Fig. 1D, left graph). For instance, in the absence of Pi, V_max_ are 0.08 ± 0.01 and 1.7 ± 0.03 μM s^−1^, at 5 and 500 nM GLS2, respectively, whereas in 20 mM Pi, V_max_ are 0.25 ± 0.03 and 2.81 ± 0.07 μM s^−1^, for equivalent concentrations. On the other hand, the analysis of the K_0.5_ values indicates an uncoupled behavior: at lower protein concentrations (5–25 nM), phosphate does not affect K_0.5,_ around 40 mM. At higher protein levels (beyond 50 nM), phosphate leads to a decreased K_0.5_, down to 10.7 ± 0.3 mM, at 500 nM GLS2 (Fig. 1D, right graph), thus improving the apparent affinity of GLS2 for l-glutamine.

Despite the increase in V_max_ that accompanies a likewise increased GLS2 concentration (Fig. 1D, left graph), the proportion is not maintained. The turnover numbers (k_cat_) in Fig. 1E, left graph, show that the maximum number of substrate conversion per second per active site substantially deteriorates as the enzyme concentrations increase (Fig. 1E). For instance, in the absence of Pi, turnover rates are 16 ± 2 s^−1^ (for 5 nM GLS2) and 3.4 ± 2 s-1 (for 500 nM GLS2), respectively, i.e., a near five-fold decrease. In 20 mM Pi, corresponding numbers are 50 ± 6 s^−1^ (for 5 nM GLS2) and 5.6 ± 0.1 s-1 (for 500 nM GLS2), a nine-fold decrease. Therefore, when enzyme efficiency is finally calculated (k_cat_/K_0.5_; Fig. 1E, right graph), it can be observed that, in the absence of phosphate (light gray), enzyme efficiency is compromised.

Indeed, as expected, phosphate increases the catalytic efficiency at all tested protein concentrations, albeit modestly, when compared to its absence. On the other hand, phosphate does not significantly alter GLS2 efficiency as protein concentration increases (Fig. 1E, right graph, dark gray).

A relative analysis, done by dividing the kinetic parameters in the presence and absence of phosphate, at equivalent protein concentrations, show that the apparent affinity for the substrate and the turnover rates (Fig. 1F, left and middle graphs) improve considerably when the enzyme is between 5 and 100 mM. Within this range, according to the respective Hill coefficients (Fig. 1C), which grow from 1 to about 2.5, the positive cooperativity results gradually in a more efficient enzyme (Fig. 1F, right graph), although efficiency peaks at already 25 nM GLS2 (according to a multivariate ANOVA). Interestingly, however, at 500 nM GLS2, when the binding sites in the tetramer may be fully occupied (n = 4), the efficiency is severely decreased (Fig. 1F, right graph) mainly because the turnover rates are slowed (Fig. 1F, middle graph).

### 2.2 The crystal structure of the human GLS2 glutaminase domain

**Fig. 2:**
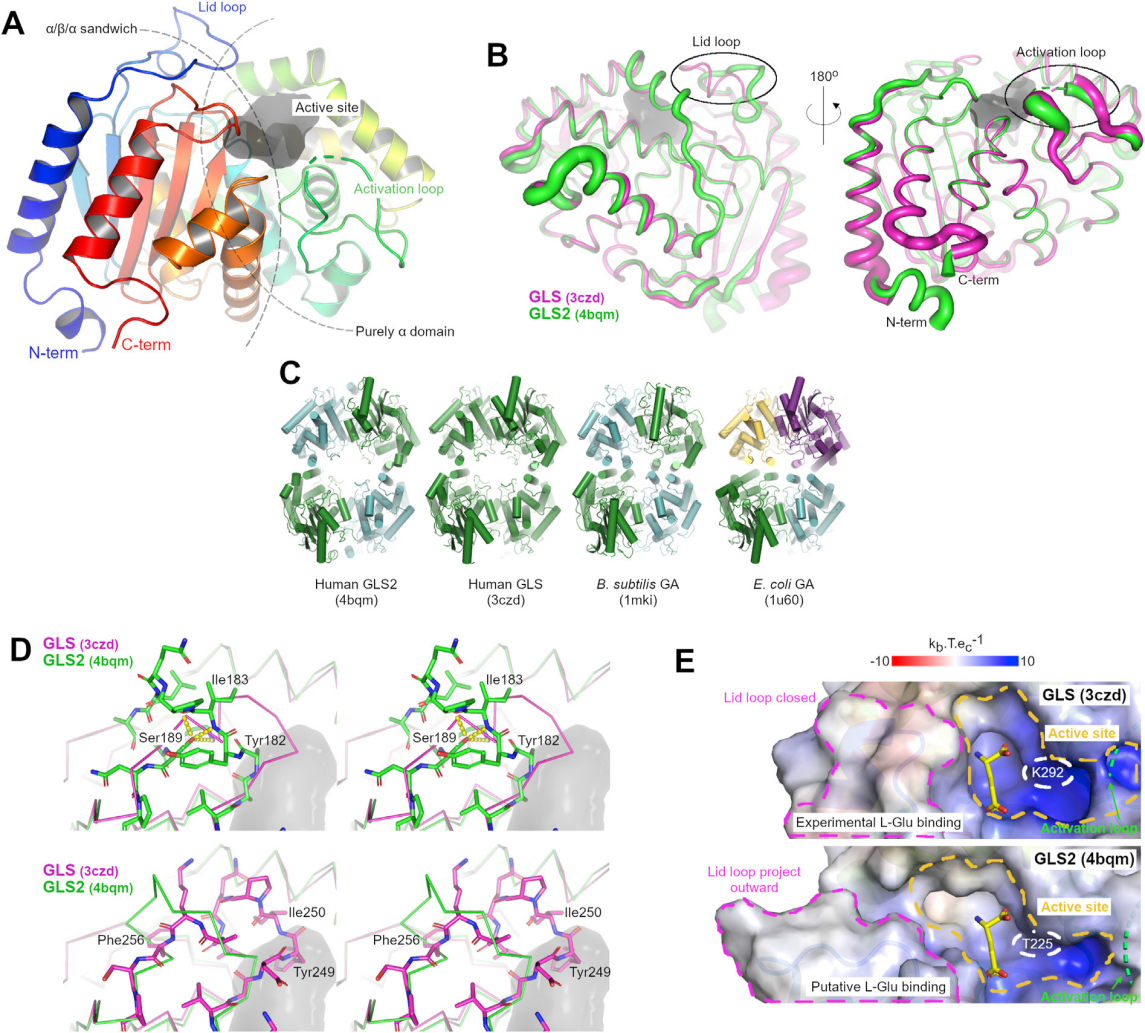
**Structural characterization of GLS2. (A)** Cartoon representation of the crystal structure of the glutaminase domain of GLS2. The solid gray surface delimits the boundaries of the active site. **(b)** GLS crystal structure (PDB: 3czd) was superposed onto GLS2 to indicate conformational differences at the lid loop and at the activation loop. Ribbon thickness is relative to the average crystallographic temperature factors. (**C**) Comparison between the tetramers of the catalytic domains of GLS2, GLS, and the glutaminases from bacteria Ybgj (*B. subtilis*) and Ybas (*E. coli*). The quaternary structure of glutaminases is highly conserved, particularly that of the tetramer. Tetramers are generated from the application of crystalline symmetry to the components of each crystal’s asymmetric unit. In this case, colors represent equivalent molecules according to crystalline symmetry. (**D**) Wall-eyed stereo view of the “lid” loop, highlighting equivalent GLS Tyr249 (magenta) and GLS2 Tyr182 (green) in different conformations because of the unique presence of Ser189 and the hydrogen bond network in GLS2 (Phe256 in GLS). (**E**) The substrate-binding cleft (delimited by the orange dashed line) is less electropositive in GLS2, particularly in the channel right to the substrate’s putative docking site; this channel may accommodate a novel stable conformation of the activation (green dashed lines), which is still missing in all crystal structures of mammalian glutaminases. A pink dashed line highlights conformational differences at the “lid” loop. The green dashed line in both panels indicated the missing activation loop.

We determined the ligand-free structure for the human GLS2 glutaminase domain at a maximum resolution of 2.2 Å (PDB 4bqm, R_factor_ of 18% and R_free_ of 20%, Table 1). The crystallized construct comprises residues Ile154 to Gly479 (Fig. 2A), common to both the LGA and GAB isoforms. The GLS2 glutaminase domain belongs to the serine-dependent beta-lactamase/transpeptidase-like superfamily of structures [29]. The active site, with a volume of approximately 500 Å^3^ (Fig. 2A), is located between an α/β/α sandwich and a purely α subdomain. As previously demonstrated for GLS glutaminases, two critical flexible loops control accessibility to this region: the “lid " region (residues Val246-Phe256 [30]) and the “activation” loop (Leu316-Phe322 [26,27,30]), which are present at different conformations or are completely disordered across the two monomers in the asymmetric unit. The novel structure presents a backbone tracing that is virtually identical to that of the human GLS glutaminase domain. The root mean square deviation in the positions of the alpha carbons of 0.6 Å (Fig. 2B) is in perfect agreement with the value expected for a sequence identity of 78.4%, according to the empirical expression derived by Chothia and Lesk [31].

**Table 1:** X-ray crystallography data collection parameters and structure refinement statistics.

Data collection	
Beamline	I03 at Diamond
Space group	H 3 2
Cell parameters a, b, c (Å)	203.6, 203.6, 99.0
Resolution range (Å)	65.83–2.18 (2.24–2.18)
Unique reflections	40642 (2830)
R_symm_ (%)	7 (53)
Completeness (%)	100 (100)
I/σ(I)	23.5 (4.4)
Average Mosaicity (°)	0.5
B-factor from Wilson Plot (Å^2^)	44.8
Monomers/AU	2
Solvent content (%)	52.2
Matthews coeff. (Å^3^/Da)	2.6
Refinement	
Resolution range (Å)	2.18–65.83
Reflections (cross-validation)	2036 (5%)
Rfactor/Rfree (%)	17.8/20.3
Rmsd from standard geometry	
Bond length (Å)	0.012
Bond angles (°)	1.435
Ramachandran plot	
Most favored (%)	96
Allowed (%)	4
Outlier (%)	0

**Fig. 3:**
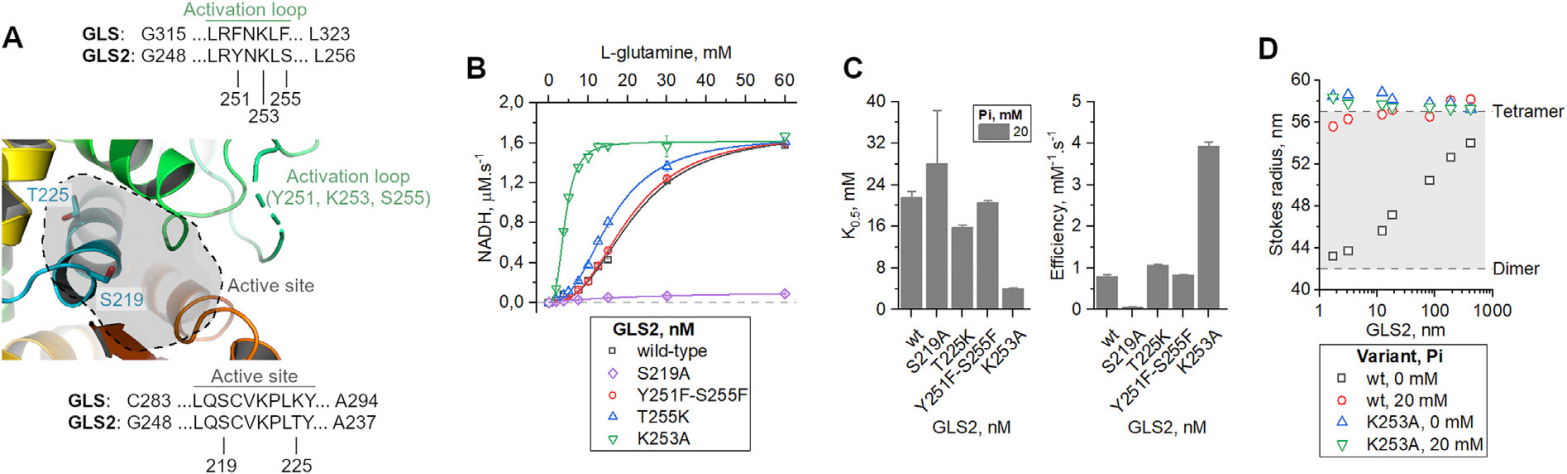
**Residues critical for GLS2 activity**. **(A)** The primary GLS2 and GLS “activation” loop sequences indicate, by numbering, the crucial amino acid substitutions for GLS and GLS2. **(B–C)** Tyr251Phe – Ser255Phe double mutation does not affect GLS2 activity; on the other hand, Thr225Lys and Lys253Ala replacements, increase active site electropositivity and modify the “activation” loop, respectively, thus increasing protein activity. As expected, the catalytic Ser219 replacement with alanine disrupted protein activity. **(D)** In the absence of phosphate, GLS2 presents a concentration-dependent oligomerization profile, shifting from dimerization to tetramerization (black squares). Phosphate addition results in a shift in the equilibrium towards higher molecular weight species, stabilizing tetramers (red circles). The high enzymatic capacity of the K235A mutant correlates with its enhanced tendency to self-assemble into tetramers, therefore suggesting the “activation” loop as a hot spot for GLS2 activity. The gray area delimits the expected Stokes radius between full-length GLS2 dimers and tetramers using the GLS structures as references.

At the quaternary level, when comparing the crystallographic structure of GLS2 and GLS with glutaminases from other organisms, such as *Bacillus subtilis* (Ybgj, PDB: 1MKI) and *Escherichia coli* (YbaS, PDB: 1U60), which are not necessarily dependent on inorganic phosphate for activation, it was noted that the tetramer (D2 symmetry dimer of dimers) is fully conserved. By applying crystalline symmetry operations inherent to each structure, we observed that they all had a tetrameric organization (Fig. 2C). The mean quadratic deviation of the tetramers’ overlap was 1.6 Å for Ybas and 1.5 Å for Ybgj.

By superposing the unbound GLS2 structure with some available GLS structures in an unbound state or bound to different elements such as glutamate, ions, and inhibitors (data not shown), we verified that the “lid” loop in GLS is always in a closed conformation, with Tyr249 facing down to the active site (Fig. 2D, PDB 3czd used as an example for GLS). In our structure, the presence of a distinct serine residue (Ser189) in GLS2 in a position corresponding to Phe256 in human GLS stabilizes a unique local rigid structure via three hydrogen bonds with adjacent residues. The hydroxyl side chain group of Ser189 contacts both the main chain amino and carboxylic groups of Ile183 and the side chain amine group of Lys188 (Fig. 2D, yellow dashes). Consequently, the phenolic hydroxyl side chain of GLS2 Tyr182 (corresponding to Tyr249 in the YIP segment in GLS) projects outward in the structure, pointing away from the active site and toward the solvent region (Fig. 2D, upper panel).

Another distinctive feature of GLS2 is the electrostatic landscape of its catalytic site (orange dashed line in Fig. 2E). The surface potential analysis of the substrate-binding cleft, based on APBS, suggests a less positively charged region than the corresponding GLS iszoyme, particularly in a channel adjacent to the right of the glutamine/glutamate binding site (Fig. 2E). The structure and sequence alignments indicated that only one residue is different in this region among the two isozymes in this region, i.e., a threonine at position 225 in GLS2, which is substituted in GLS by a lysine (Fig. 3A, and dashed white lines in Fig. 2E). Accordingly, the point mutant T225K experimentally decreased the *K*_*0.5*_ of GLS2 from 22 ± 1 mM (wild type) to 15 ± 1 mM for 100 nM protein, making it slightly more similar to GLS. We speculate that the driving factor of this increase in l-glutamine’s apparent affinity upon threonine substitution may not be the charge itself because neutrally charged amide should bind more tightly with fewer charged groups nearby. Upon closer structural inspection, this channel may accommodate the previously unseen active conformation of the so-called “activation” loop (dashed green lines in Figs. 2E, and Fig. 3A), which is still missing from all crystal structures of mammalian glutaminases determined to date. The remaining differences in the surface’s overall shape, flanking left the active site are mostly due to repositioning the “lid” loop, as described in the previous paragraph (dashed pink lines, Fig. 2E).

### 2.3 Site-directed mutagenesis reveals additional crucial residues for enzyme activity

We have previously identified the “activation” loop (Leu316-Phe322 [30], formerly referred to as the “gating” loop [26]), adjacent to the active site, as essential for filament formation and phosphate-dependent activation of the GLS isoforms [26,27]. Like most available GLS structures, the “activation” loop does not assume a preferential conformation in the ligand-free form of GLS2 (Leu249-Ser255, Fig. 3A). In GLS2, two positions (GLS2 Tyr251 and Ser255) in the “activation” loop are different from those of GLS (GLS Phe318 and Phe322, Fig. 3A), which may explain the differences in their activity profiles. However, a double-substituted GLS2 mutant (Tyr251Phe-Ser255Phe), mimicking the GLS “activation” loop, behaved similarly to wild-type GLS2, as indicated by K_0.5_ values and catalytic efficiency data (Fig. 3B and C). Here, the assays were performed at 100 nM enzyme and 20 mM phosphate, where relative efficiency is maximized (Fig. 1E). Finally, we evaluated the importance of Lys253 in GLS2. This position corresponds to Lys320 in human GLS, where a substitution to alanine led to highly active mutant GLS that was prone to form polymers [24] spontaneously. The Lys253Ala mutant has a five-fold lower K_0.5_ value than the wild type (4 ± 1 mM GLS2 with Lys253Ala and 21 mM for the wild type) and a catalytic efficiency four-fold higher (Fig. 3b-c). The Lys253Ala mutant protein also contributes to stabilizing the tetramers, as shown in Fig. 3D, maintaining GLS2 in the tetrameric form even at low concentrations and in the absence of phosphate. Altogether, the “activation” loop in GLS2 regulates tetramerization and substrate access to the active site.

## 3 Discussion

### 3.1 GLS2 is a cooperative glutaminase

While the kidney enzyme KGA initially showed Michaelian hyperbolic kinetics according to glutamine concentration, with a K_m_ of 4–5 mM [32], the liver enzyme LGA has sigmoidal kinetics, with *K*_0.5_ of 17 mM for glutamine at 50 mM Pi (reviewed by Ref. [33]). The distinguishing feature of liver glutaminase is its requirement for ammonia as an obligatory activator. More specifically, it has been shown that for human GLS2, from fresh surgical biopsy specimens, ammonia shifts K_0.5_ to lower values without changing V_max_ or sigmoidicity [14]. Similar requirements were demonstrated in intact mitochondria [13], swollen mitochondria or sonicated mitochondria [34], and with the partially purified enzyme [35].

Furthermore, the liver glutaminase’s kinetic properties are different depending on whether the enzyme is associated with the inner mitochondrial membrane or solubilized by sonication or phospholipase treatment. When membrane-associated, hepatic glutaminase has a K_m_ of 6 mM for glutamine and exhibits hyperbolic behavior according to the glutamine concentration. However, the solubilized enzyme shows a higher *K*_0.5_ for glutamine and exhibits sigmoidal kinetics [33]. As highlighted earlier, the shift to the left of the sigmoidal curve induced by ammonia allows increased activity at physiological glutamine concentrations in mitochondria [14].

In agreement with these findings, we demonstrated the GLS2 sigmoidal kinetics *in vitro*, especially at high protein concentrations and in the presence of phosphate. We further showed that the Hill coefficient change follows an increasing pattern concomitant to tetramerization, suggesting that allosteric cooperation follows. However, while cooperativity enhances the substrate’s apparent affinity, the rates of conversion are compromised, resulting in conserved efficiency throughout. This is a very different scenario than that described for GLS, since the latter is a noncooperative enzyme (i.e., hyperbolic kinetics) and relies on polymerization for a highly increased efficiency [26,27]. However, further experimentation is required to test the effects of infused ammonia in the assays with the purified recombinant protein. The protein concentrations tested in this work may be reached in the cells due to protein-protein interactions, which may direct the protein to a specific organelle or cell region. The ankyrin domains and ZB motif in the C-terminus and NRBox (nuclear receptor box) in the N-terminus of GLS2 can act as scaffolds for protein-protein interactions in mitochondria or in other organelles, as demonstrated recently for KGA [36]. They can also be involved in determining protein localization in the cell. The ZB motif binds to glutaminase-interacting protein-1, a PDZ domain-containing protein in the brain, and it can be involved in glutaminase cell localization [8,25,37].

The oligomeric properties of the liver and kidney enzymes are different. Kidney glutaminase polymerizes into higher-order species (filaments) in phosphate/borate buffer [27,38,39]. The liver enzyme appears to be a tetramer that does not require higher-order polymerization [27,40]. The maximum Hill coefficient value calculated for GLS2 was 4, and it was reached when the protein was in the tetrameric form, with or without phosphate, according to our analytical gel filtration findings. The total number of ligand binding sites is an upper bound for the experimentally determined *n* [41,42], indicating that glutamine likely has four allosteric binding sites in the tetramer. However, putative fully binding sites compromise the enzyme’s efficiency by slowing substrate turnover (Fig. 1E). To date, in addition to the active site itself, no additional binding region for l-glutamine or any other ligand has been verified for mammalian glutaminases. For instance, ammonia, which is another product of catalysis, and known to activate GLS2 [14] significantly, may directly bind to the protein at another site as it accumulates, thus contributing to the observed cooperativity. Further experimentation is required to answer these and other fundamental questions, such as why GLS2 is an allosteric enzyme and why it is so highly regulated.

Given that the tetramerization interface places four neighboring active sites in very close proximity [26], we propose that the positive cooperativity described here is due to concerted allosteric regulation among the adjacent active sites upon oligomerization, limited to tetramer formation. A more drastic improvement in the catalytic constants was observed for GLS, extending into polymers, with tetramers as the minimum repeating unit [27]. Overlaying the GLS and GLS2 crystal structures shows that the catalytic domains fold in a nearly identical fashion with few stretches of residues that differ significantly. Indeed, as we have previously demonstrated, differences in oligomerization capacity are in the N- and C-terminal portions of these two isozymes [27].

In our previous work, we described the GAC structure bound to inorganic phosphate. The phosphate ion was buried inside GAC’s highly positive active site, making polar contacts with the catalytic Ser291 and other residues in the catalytic site. Moreover, we showed that phosphate plays a central role in increasing the enzyme’s turnover rate [26]. Enzyme inhibition by glutamate has been reported for GLS isoforms [33], and we can now speculate this to be due to a more positive active site (Fig. 2D). Furthermore, Sayre and Roberts showed that the glutamate-induced inhibition of glutaminases is based on its competition with phosphate [44]. In this sense, we initially proposed that Pi competes with glutamate for the cationic active site, accelerating product exchange and simultaneously preventing enzyme inhibition as the product accumulates [26]. Later, Li and colleagues [30] described replacing the Tyr254 (present in the highly conserved YIP sequence within the “lid” loop, present at the opening of the catalytic site) to Phe in GAC, which caused a significant shift in the dose-response to inorganic phosphate (leading to a decrease in the *K*_0.5_ for this ion), indicating that disrupting the active site lid with a Tyr to Phe substitution can significantly enhance the binding of inorganic phosphate and alter the specific activity of the enzyme.

We also verified that inorganic phosphate aids in stabilizing tetramers, but not filaments; the tetrameric form in the presence of phosphate has the lowest enzyme efficiency. Activated by phosphate, GLS has Tyr254 in the “lid” loop pointing inward, and the GLS crystallographic structure showed a phosphate ion within the catalytic site [26]. Moreover, replacing this Tyr with a Phe increased GLS affinity for phosphate [30], implying that the phenolic hydroxyl group expels phosphate from this position. On the other hand, the GLS2 ″lid” loop has a stabilized conformation with Tyr182 (corresponding to Tyr249 in GLS) pointing outward to the catalytic site. Because GLS2 also binds phosphate within its catalytic site, the Tyr182 orientation may explain why GLS2 has a decreased K_0.5_ for phosphate and a higher affinity for phosphate binding. We previously demonstrated that phosphate enhances the GLS2 glutamine K_0.5_ [26] and shows that this enhancement holds for a protein concentration of 10 nM. Altogether, we speculate that the higher affinity of GLS2 for phosphate (compared to that of GLS) relates to a disturbance of the phosphate and glutamine interaction and an increase in glutamine K_0.5._ Curiously, the lowest K_0.5_ for glutamine was observed when the protein was stabilized in tetramers (at higher protein concentrations) and was enhanced by phosphate, implying that, under this condition, the disturbance-based mechanism was no longer operational.

### 3.2 The GLS2 structure is a resource for designing GLS-oriented inhibitors

Previous work has revealed a series of alkyl benzoquinones that inhibit GLS2 more strongly than they do to GLS and decreased intracellular glutaminase activity in lung, breast, and liver carcinoma cell lines [22]. GLS2 was also shown to be tumorigenic in breast cancer [23,24]. These findings validate GLS2 as a potential anticancer target.

The GLS-specific inhibitors BPTES and its more potent analog, CB-839, prevent polymer formation and GLS activation [27] by trapping the “activation” loop of GLS in a rigid open conformation [27,45]. The molecular basis of BPTES specificity for GLS was shown to involve Phe318 and Phe322 [45], which are tyrosine and serine, respectively, in GLS2. These are the only amino acid differences between the GLS and GLS2 ″activation” loops. Another “activation” loop residue essential for BPTES binding, Lys320 (based on human GLS numbering), was also shown to be critical for GLS activation [27]. Although polymer formation is not required for its activation, GLS2 activity is driven by the “activation” loop and is controlled by Lys253 (Lys320 in GLS), the replacement of which with alanine drives the protein to a tetrameric, highly activated form. Considering these findings, we established the GLS2 ″activation” loop as a hot spot for the targeted design of small-molecule inhibitors with the possibility of fine-tuning GLS/GLS2 exclusivity via the Phe318/Tyr251 and Phe322/Ser255 substitutions. Finally, the description of GLS2 as a pro-oncogenic protein has unique implications for the future development of small-molecule-oriented therapeutics targeting glutaminases in cancer.

Overall, our results show that increased levels of GLS2 result in the stabilization of tetramers and sigmoidal nonclassical Michaelis-Menten kinetics. The GLS2 ″lid” loop presents a stabilized conformation with Tyr182 pointing outwards, explaining the decreased K_0.5_ for phosphate in the catalytic site (and, consequently, phosphate inhibition). Finally, the GLS2 ″activation” loop also has a role in controlling protein activity, as shown by the Lys253Ala mutation enhancing the protein activity without driving the protein to oligomeric forms of higher order than a tetramer.

## 4 Material and methods

### 4.1 Recombinant protein production, enzymatic assay, size-exclusion serial dilution, and site-directed mutagenesis

Human GLS2 was used for crystallization purposes, while enzyme kinetics assays were performed with the mouse construct, which shares a 95% sequence identity with the human protein. Mouse GLS2 (Leu72-Val602) cloning was previously described [26]. Point mutants were generated by PCR using internal primers with the mutations and the QuickChange II XL site-directed mutagenesis kit (Agilent Technologies). The human GLS2 catalytic domain construct (NP_037399.2, Ile154-G479) was cloned into the pNIC28-Bsa4 vector and expressed in *E. coli* strain BL21(DE3)-R3-pRARE2. The cells were grown in 10 L of terrific broth medium, and the expression was induced by 0.1 mM IPTG added at 18 °C. Then, the cells were harvested and resuspended in 50 mM HEPES, pH 7.5; 500 mM NaCl; 20 mM imidazole; 5% glycerol; 0.5 mM TCEP and 1 tablet of EDTA-free protease inhibitor cocktail (Roche) in solution and disrupted by passing through a high-pressure homogenizer at 15,000 psi for 4 cycles. The soluble fraction was loaded onto Ni-NTA resin (Qiagen), and the recombinant protein (which contained a His-tag fusion on its N-terminus) was eluted with 250 mM imidazole. The eluate was then loaded onto a HiLoad 16/60 Superdex 200 (GE Healthcare) gel filtration column equilibrated with 10 mM HEPES, pH 7.5; 500 mM NaCl; 0.5 mM TCEP; and 5% glycerol. After gel filtration, the protein was concentrated using an Amicon ultrafiltration device (10 KDa cutoff; Millipore), diluted in HEPES pH 8.0 solution, and then loaded onto a HiTrap Q HP (GE Healthcare) anionic exchange column. The elution was performed with a linear gradient of NaCl from 50 mM to 2 M over 70 min. The streamlined glutaminase activity assay and the size-exclusion analysis of the serial dilution were performed as previously published, with the exception that the increased amounts (as indicated in the text) of GLS2 were used in the assays. Measurements were performed in triplicate and analyzed using GraphPad Prism 5 (GraphPad software) and Origin 8.1 (OriginLab). A general formula of a Hill equation for dose-response kinetics of the type:

$$V=V_{min}+\left(V_{max}-V_{min}\right)\frac{{\left[S\right]}^{n}}{K_{0.5}^{n}+{\left[S\right]}^{n}}$$

was used to model the curves, where *V*_*min*_ and *V*_*max*_ are the minimum and maximum reaction rates for a given protein concentration, respectively; [S] is the substrate (l-glutamine) concentration for each assay; *K*_*0.5*_ is the substrate concentration occupying one-half of the active sites; *n* is the Hill coefficient that determines the degree of cooperativity. *V*_*min*_, *V*_*max*_, *K*_*0.5*_, and *n* were freely adjusted during curve fitting.

### 4.2 Crystallization and X-ray crystallography

Crystals were grown by the sitting drop vapor diffusion method at 293 K. A sitting drop consisting of 100 nl of protein in 13 mg/ml and 50 nl well solution was equilibrated against the well solution containing 0.1 M Tris, pH 8.5; 0.2 M sodium chloride; and 25% (w/v) PEG 3350. The crystals were mounted in the presence of 25% (v/v) ethylene glycol and flash-cooled in liquid nitrogen. X-ray diffraction data were obtained with Diamond Light Source beamline I03. Data were processed using Mosflm [46] and Scala [47] software. The first set of phases was obtained by molecular replacement as implemented in Phaser [48], using the crystallographic model of the glutaminase domain of GLS (PDB code 3czd [26]). Positional and B-factor refinement cycles were performed with Refmac [49]. The manual generation of the extra portions and real-space refinement, including Fourier electron density map inspection, were performed with Coot [50]. The final model’s overall stereochemical quality and the agreement between them and the experimental data were assessed by the program Molprobity [51] and the appropriate Coot routines.

## Author contributions

The manuscript was written through the contributions of all authors. SMGD, ALBA, and WWY designed the research; IMF, JENQ, ACSB, CTR, MV, TK, CSD, and NBB conducted the experiments; SMGD, ALBA, FVD, and WWY analyzed the experimental data; SMGD, ALBA, and IMF wrote the paper. All authors have given approval to the final version of the manuscript.

## Funding sources

This work was supported by 10.13039/501100001807São Paulo State Research Foundation, FAPESP, under grants 2012/14298-9 and 2014/20673-2 (A.L.B.A.), 2014/15968-3 and 2019/16351-3 (S.M.G.D.) and fellowships 2013/05668-0 and BEPE 2012/04563-7 (I.M.F.). We acknowledge CIBFar (10.13039/501100001807FAPESP CEPID 2013/07600-3) for full support. The Structural Genomics Consortium is a registered charity (number 1097737) that receives funds from AbbVie, Boehringer Ingelheim, the Canada Foundation for Innovation, the Canadian Institutes for Health Research, Genome Canada, GlaxoSmithKline, Janssen, Lilly Canada, the 10.13039/100004336Novartis 10.13039/100005930Research Foundation, the 10.13039/501100000192Ontario Ministry of Economic Development and Innovation, Pfizer, Takeda, and the Wellcome Trust [092809/Z/10/Z].

## Declaration of competing interest

The authors declare no conflict of interest related to this work.

## References

[1] Koppenol W.H., Bounds P.L., Dang C.V. (2011). Otto Warburg’s contributions to current concepts of cancer metabolism. Nat. Rev. Canc..

[2] Wise D.R., Thompson C.B. (2010). Glutamine addiction: a new therapeutic target in cancer. Trends Biochem. Sci..

[3] Wang J.-B., Erickson J.W., Fuji R., Ramachandran S., Gao P., Dinavahi R., Wilson K.F., Ambrosio A.L.B., Dias S.M.G., Dang C.V., Cerione R.A. (2010). Targeting mitochondrial glutaminase activity inhibits oncogenic transformation. Canc. Cell.

[4] Gao P., Tchernyshyov I., Chang T.C., Lee Y.S., Kita K., Ochi T., Zeller K.I., De Marzo A.M., Van Eyk J.E., Mendell J.T., Dang C.V. (2009). C-Myc suppression of miR-23a/b enhances mitochondrial glutaminase expression and glutamine metabolism. Nature.

[5] Lukey M.J., Greene K.S., Erickson J.W., Wilson K.F., Cerione R.A. (2016). The oncogenic transcription factor c-Jun regulates glutaminase expression and sensitizes cells to glutaminase-targeted therapy. Nat. Commun..

[6] Elgadi K.M., Meguid R.A., Qian M., Souba W.W., Abcouwer S.F. (1999). Cloning and analysis of unique human glutaminase isoforms generated by tissue-specific alternative splicing. Physiol. Genom..

[7] Martín-Rufián M., Tosina M., Campos-Sandoval J.A., Manzanares E., Lobo C., Segura J.A., Alonso F.J., Matés J.M., Márquez J. (2012). Mammalian glutaminase Gls2 gene encodes two functional alternative transcripts by a surrogate promoter usage mechanism. PloS One.

[8] Márquez J., López de la Oliva A.R., Matés J.M., Segura J.A., Alonso F.J., Glutaminase (2006). A multifaceted protein not only involved in generating glutamate. Neurochem. Int..

[9] Il Chung-Bok M., Vincent N., Jhala U., Watford M. (1997). Rat hepatic glutaminase: identification of the full coding sequence and characterization of a functional promoter. Biochem. J..

[10] Campos-Sandoval J.A., López de la Oliva A.R., Lobo C., Segura J.A., Matés J.M., Alonso F.J., Márquez J. (2007). Expression of functional human glutaminase in baculovirus system: affinity purification, kinetic and molecular characterization. Int. J. Biochem. Cell Biol..

[11] Gómez-Fabre P.M., Aledo J.C., Del Castillo-Olivares A., Alonso F.J., Núñez De Castro I., Campos J.A., Marquez J. (2000). Molecular cloning, sequencing and expression studies of the human breast cancer cell glutaminase. Biochem. J..

[12] de la Rosa V., Campos-Sandoval J.A., Martín-Rufián M., Cardona C., Matés J.M., Segura J.A., Alonso F.J., Márquez J. (2009). A novel glutaminase isoform in mammalian tissues. Neurochem. Int..

[13] Verhoeven A.J., van Iwaarden J.F., Joseph S.K., MeijerR A.J. (1983). Control of rat-liver glutaminase by ammonia and pH. Eur. J. Biochem..

[14] Snodgrass P.J., Lund P. (1984). Allosteric properties of phosphate-activated glutaminase of human liver mitochondria. Biochim. Biophys. Acta Gen. Subj..

[15] Martín-Rufián M., Tosina M., Campos-Sandoval J.A., Manzanares E., Lobo C., Segura J.A., Alonso F.J., Matés J.M., Márquez J. (2012). Mammalian glutaminase Gls2 gene encodes two functional alternative transcripts by a surrogate promoter usage mechanism. PloS One.

[16] Hu W., Zhang C., Wu R., Sun Y., Levine A., Feng Z. (2010). Glutaminase 2, a novel p53 target gene regulating energy metabolism and antioxidant function. Proc. Natl. Acad. Sci..

[17] Suzuki S., Tanaka T., Poyurovsky M.V., Nagano H., Mayama T., Ohkubo S., Lokshin M., Hosokawa H., Nakayama T., Suzuki Y., Sugano S., Sato E., Nagao T., Yokote K., Tatsuno I., Prives C. (2010). Phosphate-activated glutaminase (GLS2), a p53-inducible regulator of glutamine metabolism and reactive oxygen species. Proc. Natl. Acad. Sci..

[18] Szeliga M., Albrecht J. (2015). Opposing roles of glutaminase isoforms in determining glioblastoma cell phenotype. Neurochem. Int..

[19] Szeliga M., Bogacińska-Karaś M., Różycka A., Hilgier W., Marquez J., Albrecht J. (2014). Silencing of GLS and overexpression of GLS2 genes cooperate in decreasing the proliferation and viability of glioblastoma cells. Tumor Biol..

[20] Liu J., Zhang C., Lin M., Zhu W., Liang Y., Hong X., Zhao Y., Young K.H., Hu W., Feng Z. (2014). Glutaminase 2 negatively regulates the PI3K/AKT signaling and shows tumor suppression activity in human hepatocellular carcinoma. Oncotarget.

[21] Qing G., Li B., Vu A., Skuli N., Walton Z.E., Liu X., Mayes P.A., Wise D.R., Thompson C.B., Maris J.M., Hogarty M.D., Simon M.C. (2012). ATF4 regulates MYC-mediated neuroblastoma cell death upon glutamine deprivation. Canc. Cell.

[22] Lee Y.Z., Yang C.W., Chang H.Y., Hsu H.Y., Chen I.S., Chang H.S., Lee C.H., chyi Lee J., Kumar C.R., Qiu Y.Q., Chao Y.S., Lee S.J. (2014). Discovery of selective inhibitors of Glutaminase-2, which inhibit mTORC1, activate autophagy and inhibit proliferation in cancer cells. Oncotarget.

[23] Dias M.M., Adamoski D., dos Reis L.M., Ascenção C.F.R., de Oliveira K.R.S., Mafra A.C.P., da Silva Bastos A.C., Quintero M., Cassago C. de G., Ferreira I.M., Fidelis C.H.V., Rocco S.A., Bajgelman M.C., Stine Z., Berindan-Neagoe I., Calin G.A., Ambrosio A.L.B., Dias S.M.G. (2020). GLS2 is protumorigenic in breast cancers. Oncogene.

[24] Lukey M.J., Cluntun A.A., Katt W.P., chong M., Lin J., Druso J.E., Ramachandran S., Erickson J.W., Le H.H., Wang Z.E., Blank B., Greene K.S., Cerione R.A. (2019). Liver-type glutaminase GLS2 is a druggable metabolic node in luminal-subtype breast cancer. Cell Rep..

[25] Pasquali C.C., Islam Z., Adamoski D., Ferreira I.M., Righeto R.D., Bettini J., Portugal R.V., Yue W.W.Y., Gonzalez A., Dias S.M.G., Ambrosio A.L.B. (2017). The origin and evolution of human glutaminases and their atypical C-terminal ankyrin repeats. J. Biol. Chem..

[26] Cassago A., Ferreira A.P.S., Ferreira I.M., Fornezari C., Gomes E.R.M., Greene K.S., Pereira H.M., Garratt R.C., Dias S.M.G., Ambrosio A.L.B. (2012). Mitochondrial localization and structure-based phosphate activation mechanism of Glutaminase C with implications for cancer metabolism. Proc. Natl. Acad. Sci..

[27] Ferreira A.P.S., Cassago A., De Almeida Gonçalves K., Dias M.M., Adamoski D., Ascenção C.F.R., Honorato R.V., De Oliveira J.F., Ferreira I.M., Fornezari C., Bettini J., Oliveira P.S.L., Leme A.F.P., Portugal R.V., Ambrosio A.L.B., Dias S.M.G. (2013). Active glutaminase C self-assembles into a supratetrameric oligomer that can be disrupted by an allosteric inhibitor. J. Biol. Chem..

[28] Katt W.P., Lukey M.J., Cerione R.A. (2017). A tale of two glutaminases: homologous enzymes with distinct roles in tumorigenesis. Future Med. Chem..

[29] El-Gebali S., Mistry J., Bateman A., Eddy S.R., Luciani A., Potter S.C., Qureshi M., Richardson L.J., Salazar G.A., Smart A., Sonnhammer E.L.L., Hirsh L., Paladin L., Piovesan D., Tosatto S.C.E., Finn R.D. (2019). The Pfam protein families database in 2019. Nucleic Acids Res..

[30] Li Y., Erickson J.W., Stalnecker C.A., Katt W.P., Huang Q., Cerione R.A., Ramachandran S. (2016). Mechanistic basis of glutaminase activation. J. Biol. Chem..

[31] Chothia C., Lesk A.M. (1986). The relation between the divergence of sequence and structure in proteins. EMBO J..

[32] Klingman J.D., Handler P. (1958). Partial purification and properties of renal glutaminase. J. Biol. Chem..

[33] Curthoys N.P., Watford M. (1995). Regulation of glutaminase activity and glutamine metabolism. Annu. Rev. Nutr..

[34] McGivan J.D., Bradford N.M. (1983). Characteristics of the activation of glutaminase by ammonia in sonicated rat liver mitochondria. BBA - Gen. Subj..

[35] Patel M., McGivan J.D. (1984). Partial purification and properties of rat liver glutaminase. Biochem. J..

[36] de Guzzi Cassago C.A., Dias M.M., Pinheiro M.P., Pasquali C.C., Bastos A.C.S., Islam Z., Consonni S.R., de Oliveira J.F., Gomes E.M., Ascenção C.F.R., Honorato R., Pauletti B.A., Indolfo N.D.C., Filho H.V.R., de Oliveira P.S.L., Figueira A.C.M., Paes Leme A.F., Ambrosio A.L.B., Dias S.M.G. (2018). Glutaminase affects the transcriptional activity of peroxisome proliferator-activated receptor γ (PPARγ) via direct interaction. Biochemistry.

[37] Olalla L., Gutiérrez A., Jiménez A.J., López-Téllez J.F., Khan Z.U., Pérez J., Alonso F.J., de la Rosa V., Campos-Sandoval J.A., Segura J.A., Aledo J.C., Márquez J. (2008). Expression of the scaffolding PDZ protein glutaminase-interacting protein in mammalian brain. J. Neurosci. Res..

[38] Olsen B.R., Svenneby G., Kvamme E., Tveit B., Eskeland T. (1970). Formation and ultrastructure of enzymically active polymers of pig renal glutaminase. J. Mol. Biol..

[39] Godfrey S., Kuhlenschmidt T., Curthoys P. (1977). Correlation between activation and dimer formation of rat renal phosphate-dependent glutaminase. J. Biol. Chem..

[40] Huang Y.Z., Knox W.E. (1976). A comparative study of glutaminase isozymes in rat tissues. Enzyme.

[41] Stefan M.I., Le Novère N. (2013). Cooperative binding. PLoS Comput. Biol..

[42] Prinz H. (2010). Hill coefficients, dose-response curves and allosteric mechanisms. J. Chem. Biol..

[44] Sayre F.W., Roberts E. (1958). Preparation and some properties of a phosphate-activated glutaminase from kidneys. J. Biol. Chem..

[45] Delabarre B., Gross S., Fang C., Gao Y., Jha A., Jiang F., Song J J., Wei W., Hurov J.B. (2011). Full-length human glutaminase in complex with an allosteric inhibitor. Biochemistry.

[46] Battye T.G.G., Kontogiannis L., Johnson O., Powell H.R., Leslie A.G.W. (2011). iMOSFLM : a new graphical interface for diffraction-image processing with MOSFLM. Acta Crystallogr. Sect. D Biol. Crystallogr..

[47] Evans P. (2006). Scaling and assessment of data quality. Acta Crystallogr. Sect. D Biol. Crystallogr..

[48] McCoy A.J., Grosse-Kunstleve R.W., Adams P.D., Winn M.D., Storoni L.C., Read R.J. (2007). Phaser crystallographic software. J. Appl. Crystallogr..

[49] Murshudov G.N., Vagin A.A., Dodson E.J. (1997). Refinement of macromolecular structures by the maximum-likelihood method. Acta Crystallogr. Sect. D Biol. Crystallogr..

[50] Emsley P., Lohkamp B., Scott W.G., Cowtan K. (2010). Features and development of Coot. Acta Crystallogr. Sect. D Biol. Crystallogr..

[51] Chen V.B., Arendall W.B., Headd J.J., Keedy D.A., Immormino R.M., Kapral G.J., Murray L.W., Richardson J.S., Richardson D.C. (2010). MolProbity: all-atom structure validation for macromolecular crystallography. Acta Crystallogr. Sect. D Biol. Crystallogr..

